# Optimal Triage Test Characteristics to Improve the Cost-Effectiveness of the Xpert MTB/RIF Assay for TB Diagnosis: A Decision Analysis

**DOI:** 10.1371/journal.pone.0082786

**Published:** 2013-12-18

**Authors:** Anna H. van’t Hoog, Frank Cobelens, Anna Vassall, Sanne van Kampen, Susan E. Dorman, David Alland, Jerrold Ellner

**Affiliations:** 1 Amsterdam Institute for Global Health and Development (AIGHD), Amsterdam, The Netherlands; 2 Department of Global Health, Academic Medical Centre, University of Amsterdam, Amsterdam, The Netherlands; 3 Social and Mathematical Epidemiology Group (SAME), Department of Global Health and Development, London School of Hygiene and Tropical Medicine, London, United Kingdom; 4 KNCV Tuberculosis Foundation, The Hague, The Netherlands; 5 Johns Hopkins University School of Medicine, Baltimore, Maryland, United States of America; 6 New Jersey Medical School-UMDNJ, Department of Medicine, Newark, New Jersey, United States of America; 7 Boston University School of Medicine and Boston Medical Center, Boston, Massachusetts, United States of America; California Department of Public Health, United States of America

## Abstract

**Background:**

High costs are a limitation to scaling up the Xpert MTB/RIF assay (Xpert) for the diagnosis of tuberculosis in resource-constrained settings. A triaging strategy in which a sensitive but not necessarily highly specific rapid test is used to select patients for Xpert may result in a more affordable diagnostic algorithm. To inform the selection and development of particular diagnostics as a triage test we explored combinations of sensitivity, specificity and cost at which a hypothetical triage test will improve affordability of the Xpert assay.

**Methods:**

In a decision analytical model parameterized for Uganda, India and South Africa, we compared a diagnostic algorithm in which a cohort of patients with presumptive TB received Xpert to a triage algorithm whereby only those with a positive triage test were tested by Xpert.

**Findings:**

A triage test with sensitivity equal to Xpert, 75% specificity, and costs of US$5 per patient tested reduced total diagnostic costs by 42% in the Uganda setting, and by 34% and 39% respectively in the India and South Africa settings. When exploring triage algorithms with lower sensitivity, the use of an example triage test with 95% sensitivity relative to Xpert, 75% specificity and test costs $5 resulted in similar cost reduction, and was cost-effective by the WHO willingness-to-pay threshold compared to Xpert for all in Uganda, but not in India and South Africa. The gain in affordability of the examined triage algorithms increased with decreasing prevalence of tuberculosis among the cohort.

**Conclusions:**

A triage test strategy could potentially improve the affordability of Xpert for TB diagnosis, particularly in low-income countries and with enhanced case-finding. Tests and markers with lower accuracy than desired of a diagnostic test may fall within the ranges of sensitivity, specificity and cost required for triage tests and be developed as such.

## Introduction

New diagnostics could substantially reduce global TB incidence [1], [2]. Nucleic acid amplification tests (NAAT) have higher sensitivity than smear microscopy [3], [4], and have high specificity. The Xpert MTB/RIF assay [5] (Cepheid, Sunnyvale, CA, USA), hereafter referred to as Xpert, is the first NAAT endorsed by World Health Organization (WHO) for widespread use [6], [7]. Xpert can confirm the presence of *M tuberculosis* and identify rifampicin resistance-conferring mutations within 2 hours, and increases case detection by one third compared to smear-microscopy [4], [8]. In economic evaluations the use of Xpert as an addition to, or a replacement of sputum smear microscopy in routine clinical settings in high-TB burden countries was cost-effective by the WHO Choosing Interventions that are Cost-Effective (WHO-CHOICE) initiative’s willingness to pay (WTP) threshold [9], [10]. This threshold considers interventions to be cost-effective if the incremental cost to avert an additional disability-adjusted life year (DALY) is less than the country per-capita gross domestic product (GDP) [11], [12].

However, the Xpert technology requires basic laboratory infrastructure, has relatively high cost compared to microscopy, and does not meet all specifications of a truly point-of-care (POC) TB diagnostic test [8], [13]. WHO therefore recommends Xpert as the initial diagnostic test in individuals with presumed MDR-TB or HIV-associated TB, and conditionally in settings where MDR and/or HIV is of lesser concern given the resource implications [7], [14]. The affordability of scaling up Xpert is a concern in many high TB burden countries in Africa and Asia, where the costs to test all persons with presumed TB in routine clinical practice with Xpert requires 20–80% of current TB spending [15]. Further, as Xpert is applied to populations with low TB prevalence, cost-effectiveness rapidly decreases due to high per-diagnosis cost [9]. This may preclude scale-up for use in improved case finding through screening, i.e. a systematic effort to identify unrecognized disease through targeting groups in which TB prevalence is lower [16], [17] than among patients with prolonged TB symptoms reporting to health facilities in high TB incidence countries [18].

A cheaper POC TB diagnostic test with optimized sensitivity and specificity therefore remains a major objective of research and development [13], [19]. Some of the available technology or biomarkers [19] that do not meet the specifications of an optimized diagnostic POC may however be modifiable into a triage test, to be used in a diagnostic algorithm [20], to select patients with increased probability of having TB for confirmatory testing by Xpert. Such a ‘triage algorithm’ could potentially reduce diagnostic cost and thus be more affordable than offering Xpert to all persons with presumptive TB. For a non-invasive triage test with high sensitivity, the cost-effectiveness of a triage algorithm is mainly a trade-off between specificity, which determines the number of persons requiring confirmatory testing by Xpert, and cost. In order to guide the development and selection of potential triage tests we expanded a decision analytical model [9] with the objective to explore combinations of sensitivity, specificity and cost of a hypothetical triage test at which a triage algorithm is more cost-effective than Xpert for all persons with presumptive TB.

## Methods

The model was parameterized with data from a demonstration study of Xpert in three epidemiological and economic settings: India (low HIV prevalence, low MDR prevalence), Uganda (high HIV prevalence, low MDR prevalence), and South Africa (high HIV prevalence, moderately high MDR prevalence) [4] and follows a cohort of 10 000 individuals with presumptive TB who require diagnostic testing through the diagnostic and treatment pathway, estimating test and treatment costs and health gains (DALYs averted, TB cases detected) [9]. The analysis was conducted from a TB program perspective.

**Figure 1:**
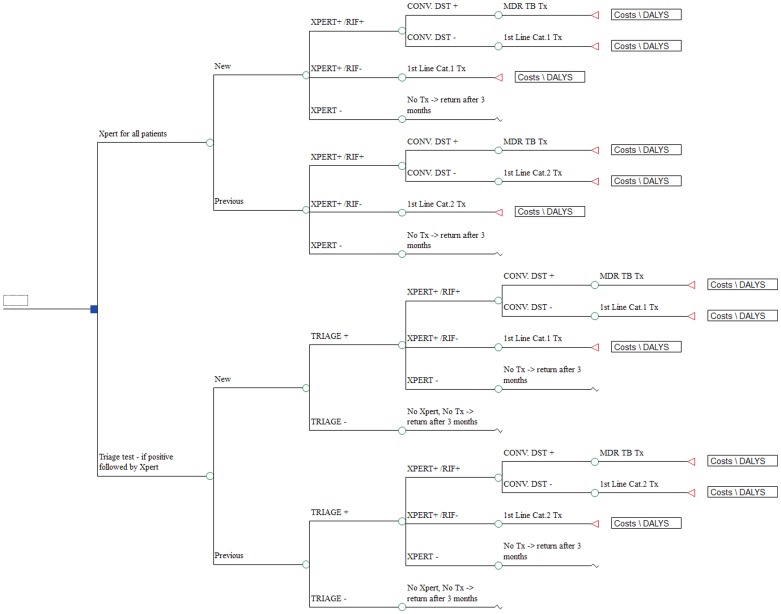
Simplified schematic presentation of the two pathways in the model.

We compared two diagnostic pathways (Figure 1), the Xpert-for-all algorithm and the triage algorithm. In the Xpert-for-all algorithm, a single sputum specimen was tested by Xpert for all persons in the cohort. In the triage algorithm, all persons were tested with a hypothetical triage test, and if positive were subsequently tested by Xpert on a single sputum specimen. In both algorithms, TB cases who remained undiagnosed due to negative results of Xpert or the triage test, respectively, return for re-testing after three months unless they die or self-cure within that time. The sensitivity of the triage test is hereon referred to as the sensitivity relative to Xpert, i.e. the proportion of cases in the cohort detectable by Xpert that test positive by the triage test. In the primary analysis the simplifying assumption was made that the proportion of Xpert-detectable cases identified by the triage test was the same in HIV-infected and HIV-uninfected TB cases. The sensitivity and specificity of Xpert was as observed in the demonstration study (all sites combined [4]) relative to sputum culture as the reference standard and was stratified by HIV- and smear-status (Table S1).

We measure and compare the effectiveness of each alternative algorithm using disability adjusted life years (DALYs) averted, a measure used in the estimation of the global burden of disease [21].

For a triage algorithm to be considered by policy makers it needs to be either as effective in identifying TB cases, but less costly than Xpert-for-all; or if less effective, the gain in cost reduction needs to be sufficiently high to make any loss in effectiveness acceptable (Figure S1). In this latter case it is relevant to report incremental cost effectiveness ratios (ICERs). These describe how much it costs to avert an additional DALY using Xpert-for-all compared to triage. If the ICER of the Xpert-for-all algorithm is higher than the amount policy makers are prepared to pay (willingness to pay (WTP) threshold) then the triage algorithm is reported as being cost-effective. According to the WHO-CHOICE criterion, WTP thresholds for highly cost-effective interventions were $1,489 for India, $8,070 for South Africa, and $487 for Uganda in 2011 [22], although lower thresholds have been suggested [12].

We therefore began by examining the characteristics of triage test with 100% sensitivity relative to Xpert, and determined the combinations of triage test specificity and test cost that result in lower total diagnostic cost of the triage algorithm compared to the Xpert algorithm. To illustrate the results and present numerical values of cost and effectiveness, we chose an hypothetical triage test example with 75% specificity and per-patient test cost of $5 (example 1).

Our second analysis explored triage tests with lower sensitivity, in which we estimated ICERs for Xpert-for-all compared to triage algorithms with different combinations of triage test sensitivity, specificity and cost. Our main purpose was to examine the relationship between per-patient triage test cost and specificity for lower levels of sensitivity. As in the primary analysis we used two-way deterministic sensitivity analysis and varied the values of cost and specificity, but now for pre-set levels of sensitivity. Here we present numerical values of cost and effectiveness of hypothetical triage test examples with sensitivity of 95% (example 2), and 85% (example 3). As illustrations we choose 75% specificity for example 2, and 85% for example 3, in the range of what can be achieved by e.g. chest radiography [23]. Following economic evaluation convention the numerical values of cost and effectiveness of these hypothetical examples are presented first, followed by the results of the sensitivity analyses that were our main interest.

We maintained key input parameters from the published model [9] but changed the following: we assumed 5% prevalence of smear-positive TB, reflecting a situation of enhanced case finding among patients attending a health facility, for instance considering all patients with a cough of any duration [16], [17]. All local costs were reported in 2011 US$ and converted using the average exchange rate for 2011 (imfstatext.imf.org). Diagnostic costs were based on costing studies conducted at the demonstration sites. The Xpert per-person test cost included costs for equipment, maintenance, labour and overheads that are country specific and were maintained from the original model [9], but were adjusted to accommodate the US$9.98 subsidized cartridge cost [24] resulting in a per-person test cost ranging from $14.01 in India to $19.23 in Uganda (Table S1). The triage test cost was also assumed to be a per-person test cost that combined cost to acquire, apply and maintain the triage test. In the examples we choose $5 per-person triage test cost as a conservative estimate. For rapid diagnostic tests diagnosing other infectious diseases in resource-limited settings, per-person test cost between $1 and $5 have been reported [25]–[27].

We conducted several sensitivity analyses to explore the effect on the cost-effectiveness of the Xpert-for-all compared to the triage algorithm of changes in TB prevalence and HIV prevalence among the cohort of patients with presumptive TB, changes in the cost of Xpert, and of the assumption that triage test sensitivity is relative to Xpert rather than to culture. We also investigated the effect on cost-effectiveness if the sensitivity of a triage test would be higher or lower in HIV-infected patients compared to HIV-negative patients, and if HIV-infected patients would have clinical diagnosis following (i) a negative Xpert result; (ii) a negative triage test result, using data on sensitivity, specificity and cost of X-ray and/or antibiotic trial taken from the demonstration study [4] (Table S1). We also explored the effect of considerably higher cost for MDR-TB treatment in South Africa [28]. Finally we conducted a probabilistic sensitivity analysis using Monte Carlo simulations to explore the effect of uncertainty around all parameter estimates. Where it was not clear whether an ICER was above the willingness to pay threshold, we produced acceptability curves. These illustrate the probability that an intervention is cost-effective given the uncertainty around any parameter estimates.

We used TreeAge (TreeAge Software, Inc., Williamstown MA, USA) for model construction and analyses, and transferred the model output to MS-Excel (Microsoft Corp, Seattle WA, USA) for further analysis. Ethical approval was not sought, as only secondary data were used.

## Results

**Figure 2:**
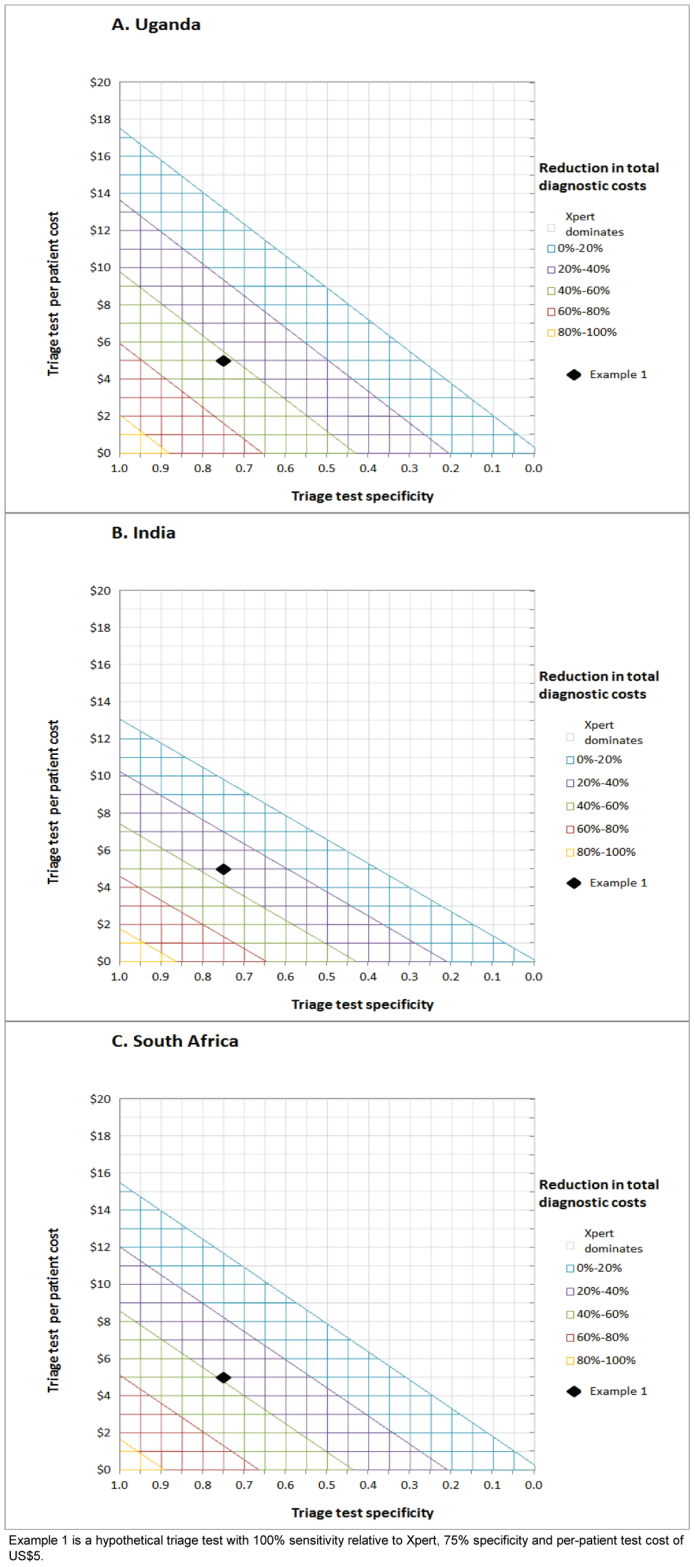
Combinations of cost and specificity of a triage test with 100% relative sensitivity. These combinations result in equal or reduced diagnostic cost of a triage pathway, compared to Xpert on all persons with presumptive TB. Panel 2A shows the Uganda setting, 2B the India setting and 2C the South African setting.

A triage test with a relative sensitivity of 100%, a specificity of 75% and per-patient test cost of $5 reduced cohort diagnostic costs for Uganda by 42% (Table 1, example 1). For India and South Africa the reductions in diagnostic costs were 34% and 39%, respectively. The reductions of total diagnostic and treatment costs combined were 23% for Uganda, 16% for India, and 11% for South Africa where the cost for TB and MDR treatment comprise a greater proportion of the total cost. The proportional reduction in diagnostic costs for other combinations of triage test specificity and per-patient test cost are shown in Figures 2A-C: e.g. more than 60% reduction could be achieved in all three settings for a triage test with 100% relative sensitivity, 85% specificity and $2 per-patient test cost.

**Table 1:** Case detection, total cohort cost and costs per patient diagnosed for Xpert-for-all and 3 example triage pathways.

Algorithm and scenarios	Cohort	Number of persons in Cohort with TB	Total TB cases detected	% of TB cases	Cohort Diagnostic Costs (US$2011)	Reduction in cohort diagnostic cost	Diagnostic cost per TB case detected (US$ 2011)	Treatment Costs (US$ 2011)	Total Costs ($2011)	Reduction in total cost	Total cost per TB case detected (US$ 2011)
**UGANDA**											
**Xpert for all persons with presumptive TB**											
	Tuberculosis (all)	959	912	95%	195294	Baseline	214	213602	408895	Baseline	448
	No Tuberculosis	9041									
	MDR TB	18	16	90%							
**Triage example 1.** 100% sensitivity, 75% specificity, cost $5; if positive followed by Xpert.											
	Tuberculosis (all)	959	912	95%	112368	42%	123	200643	313012	23%	343
	MDR TB	18	16	90%							
**Triage example 2.** 95% sensitivity, 75% specificity, cost $5											
	Tuberculosis (all)	959	895	93%	112204	43%	125	197046	309250	24%	345
	MDR TB	18	16	88%							
**Triage example 3.** 85% sensitivity, 85% specificity, cost $5											
	Tuberculosis (all)	959	853	89%	94299	52%	111	186160	280460	31%	329
	MDR TB	18	15	84%							
**INDIA**											
**Xpert for all persons with presumptive TB**											
	Tuberculosis (all)	695	684	99%	141835	Baseline	207	273885	415720	Baseline	607
	No Tuberculosis	9305									
	MDR TB	36	33	93%							
**Triage example 1.** 100% sensitivity, 75% specificity, cost $5; if positive followed by Xpert.											
	Tuberculosis (all)	695	684	99%	93458	34%	137	255064	348521	16%	509
	MDR TB	36	33	93%							
**Triage example 2.** 95% sensitivity, 75% specificity, cost $5											
	Tuberculosis (all)	695	677	97%	93487	34%	138	252267	345754	17%	511
	MDR TB	36	33	92%							
**Triage example 3.** 85% sensitivity, 85% specificity, cost $5											
	Tuberculosis (all)	695	653	94%	80373	43%	123	241119	321493	23%	492
	MDR TB	36	32	89%							
**SOUTH AFRICA**											
**Xpert for all persons with presumptive TB**											
	Tuberculosis (all)	956	910	95%	174726	Baseline	192	811040	985767	Baseline	1083
	No Tuberculosis	9044									
	MDR TB	92	82	90%							
**Triage Example 1.** 100% sensitivity, 75% specificity, cost $5; if positive followed by Xpert.											
	Tuberculosis (all)	956	910	95%	107272	39%	118	771254	878526	11%	965
	MDR TB	92	82	90%							
**Triage example 2.** 95% sensitivity, 75% specificity, cost $5											
	Tuberculosis (all)	956	893	93%	107103	39%	120	757378	864481	12%	968
	MDR TB	92	81	88%							
**Triage example 3.** 85% sensitivity, 85% specificity, cost $5											
	Tuberculosis (all)	956	851	89%	91207	48%	107	716733	807940	18%	949
	MDR TB	92	77	84%							

% prevalence of smear-positive TB. For a cohort of 10 000 persons with presumptive TB with 5

=  multi-drug resistant tuberculosis. MDR TB

=  GeneXpert MTB/RIF assay. Xpert

For triage tests with lower sensitivity (hypothetical examples 2 and 3), the percentage cases detected and effectiveness of the triage algorithm are shown in Tables 1 and 2 respectively. For example 2, a triage test with 95% sensitivity, 75% specificity and $5 cost, the diagnostic costs and cost reductions to be achieved were almost the same as for example 1. The ICER of the Xpert-for-all algorithm, compared to a triage algorithm with triage test example 2 was $491 for Uganda, i.e. one pays $491 for each additional DALY that is averted by employing the Xpert-for-all rather than the triage algorithm. At a WTP of $487 the probability that Xpert-for-all is cost-effective is 49% (Figure S4) and the triage algorithm may be the preferred choice. For India the ICERs of the Xpert-for-all algorithm compared to triage example 2 (95% relative sensitivity) was $662, less than half of the GDP, and for South Africa the ICER was $669, less than 10% of the GDP, for both countries suggesting that compared to this triage algorithm Xpert-for-all is cost-effective by the WHO-CHOICE WTP threshold.

**Table 2:** Total cohort cost, DALYS, cost per DALY and ICER for Xpert on all compared to triage pathways as the base case.

Country	Algorithm and scenarios	Total Costs ($2011)	Incremental Costs	Total DALYS	Incremental DALYs averted	Cost per DALY averted	ICER of Xpert-forall, compared to the triage pathway example	Monte Carlo Simulation ICER, Median (2.5, 97.5)*
**Uganda**	**Triage; if positive followed by Xpert.**							
	Example 2. 95% sensitivity, 75% specificity, cost $5	309250		12102		26	base case	
	Xpert for all persons with presumptive TB	408895	99646	12306	203	33	491	487 (392, 597)
	Example 3. 85% sensitivity, 85% specificity, cost $5	280460		11566		24	base case	
	Xpert for all persons with presumptive TB	408895	128436	12306	740	33	174	173 (144, 206)
**India**	**Triage; if positive followed by Xpert.**							
	Example 2. 95% sensitivity, 75% specificity, cost $5	345754		9904		35	base case	
	Xpert for all persons with presumptive TB	415720	69966	10009	106	42	662	672 (541, 833)
	Example 3. 85% sensitivity, 85% specificity, cost $5	321493		9568		34	base case	
	Xpert for all persons with presumptive TB	415720	94228	10009	442	42	213	216 (179, 260)
**South Africa**	**Triage; if positive followed by Xpert.**							
	Example 2. 95% sensitivity, 75% specificity, cost $5	864481		10176		85	base case	
	Xpert for all persons with presumptive TB	985767	121286	10351	175	95	695	695 (575, 836)
	Example 3. 85% sensitivity, 85% specificity, cost $5	807940		9719		83	base case	
	Xpert for all persons with presumptive TB	985767	177827	10351	632	95	281	282 (240, 330)

% prevalence of smear-positive TB. For a cohort of 10 000 persons with presumptive TB with 5

= incremental cost-effectiveness ratio. ICER

= disability adjusted life year. DALY

=  multi-drug resistant. MDR

=  GeneXpert MTB/RIF assay. Xpert

Fixed values for the sensitivity, specificity and cost of the triage test.

**Figure 3:**
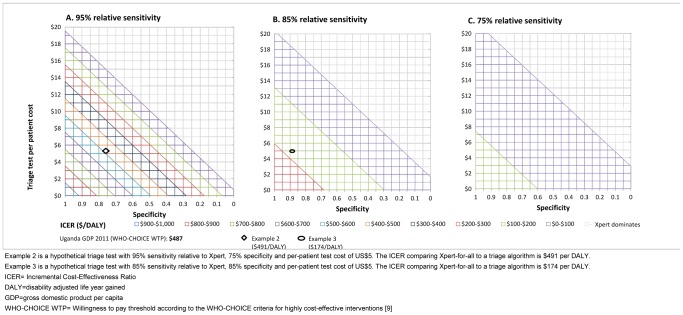
Incremental Cost-Effectiveness Ratios (ICERs) of Xpert on all persons with presumptive TB compared to triage algorithms for various sensitivity, specificity and cost combinations of a triage test. The figure shows the Uganda setting.

If the cost-effectiveness ceiling were lower than the WHO-CHOICE WTP, certain combinations of specificity and cost would make a triage test with lower sensitivity attractive. This is illustrated in Figures 3A-C for Uganda: Assuming a triage per-person test cost of $5, the ICER of Xpert-for-all compared to a triage algorithm with 85% sensitivity and 85% specificity of the triage test (example 3) was $174 per DALY averted (Figure 3B). If the sensitivity were 95% (Figure 3A), the same ICER ($174 per DALY) is expected if specificity were 40%, while many combinations of cost and specificity would result in higher ICERs. If a triage test had 75% sensitivity the ICERs were all below $174, regardless of cost and specificity (Figure 3C). Similar trends for India and South Africa are illustrated in the supplement (Figures S2-S3).

**Figure 4:**
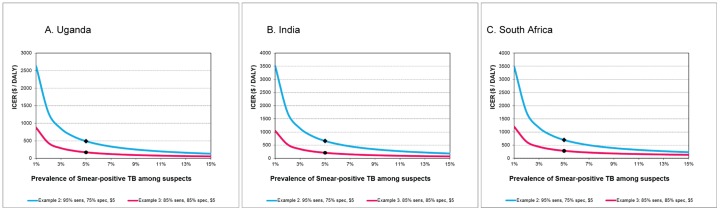
Sensitivity analyses. The effect of varying the prevalence of TB among persons with presumptive TB on the Incremental Cost-Effectiveness ratios (ICERs) for Xpert compared to triage pathways, with triage tests examples with sensitivity and specificity of 95%/75% and 85%/85% respectively, for the Uganda (4A), India (4B) and South Africa (4C) setting.

In sensitivity analyses the ICERs of Xpert-for-all compared to a triage algorithm rapidly increased at lower TB prevalence, especially for prevalence of smear-positive TB <3%, and decreased if the prevalence of smear-positive TB increased (Figure 4A-C). Assuming 2% prevalence of smear-positive TB and a triage test with 95% sensitivity and 75% specificity (example 2), the ICER was $1,292 for Uganda. Assuming 15% prevalence of smear-positive TB and a triage test with 85% sensitivity and 85% specificity (example 3) the ICER was $57 only.

Additional sensitivity analyses are shown in Table 3. If the per-patient test cost of Xpert reduced by $5, the ICERs of the Xpert-for-all algorithm compared to a triage algorithm were lower, $319 per DALY averted for example 2 and $120 DALY averted for example 3 for Uganda (Table 3). Assuming lower triage test sensitivity in HIV-positive compared to HIV-negative patients reduced the ICERs of Xpert-for-all in the two countries with high HIV prevalence (Table 3). Assuming the reverse, higher sensitivity in HIV-positive patients, made a triage algorithm more favourable, explained by greater mortality from undetected TB in HIV-positive patients. The addition of clinical diagnosis after a negative Xpert in HIV-positive patients (Table S2) increased the total cost of the Xpert-for-all algorithm by 18%, and of triage algorithms by 4–6% for Uganda. Adding clinical diagnosis also after a negative triage test increased the total cost of the triage algorithm by 24–27%, partly due to treatment cost for an increased number of TB cases diagnosed empirically without bacteriologic confirmation, which has low specificity.

**Table 3:** Sensitivity analyses.

Assumption	Triage test example	ICER of Xpert-for-all compared to triage algorithm (Costs per DALY, 2011 US$)		
		Uganda	India	South Africa
Primary estimate	2. 95% sensitivity, 75% specificity, cost $5*	491	662	695
	3. 85% sensitivity, 85% specificity, cost $5	174	213	281
Xpert cartridge price changes –/+ $5	2. 95% sensitivity, 75% specificity, cost $5	319–662	329–996	495–894
	3. 85% sensitivity, 85% specificity, cost $5	120–227	123–304	218–344
HIV-prevalence –/+ 25% in high HIV settings	2. 95% sensitivity, 75% specificity, cost $5	551–442		803–614
	3. 85% sensitivity, 85% specificity, cost $5	184–165		301–266
Triage test sensitivity relative to culture and independent of Xpert sensitivity	2. 95% sensitivity, 75% specificity, cost $5	624	846	867
	3. 85% sensitivity, 85% specificity, cost $5	203	244	321
Triage test sensitivity lower in HIV-positive compared to HIV-negative patients	2. 90% in HIV+, 100% in HIV–, 75% spec, $5	326	23,380	467
	3. 75% in HIV+, 95% in HIV–, 85% spec, $5	136	760	230
Triage test sensitivity higher in HIV-positive compared to HIV-negative patients	2. 100% in HIV+, 90% in HIV–, 75% spec, $5	723	295	1104
	3. 95% in HIV+, 75% in HIV–, 75% spec, $5	190	114	312
Increase upper range of MDR treatment in South Africa to $17,164 [28]	2. 95% sensitivity, 75% specificity, cost $5			753
	3. 85% sensitivity, 85% specificity, cost $5			336

The effect of assumptions on the ICER of Xpert-for-all compared to triage pathway with different triage test examples.

per-patient test cost including cost to acquire, apply and maintain the test.

= incremental cost-effectiveness ratio. ICER

= disability adjusted life year. DALY

=  multi-drug resistant. MDR

=  GeneXpert MTB/RIF assay. Xpert

+  = HIV positive; HIV–  =  HIV negative; spec = specificity. HIV

## Discussion

In this decision analytical modeling study we explored the concept of triaging patients for confirmatory testing by Xpert and demonstrated that triage tests with high sensitivity but modest specificity could improve the affordability of Xpert in current clinical settings. We show a range of (combinations of) sensitivity, specificity and cost within which a potential triage test would result in a considerable reduction of total diagnostic costs compared to Xpert for all patients with presumptive TB. As hypothetical examples, for a triage test that would be as sensitive as Xpert, approximately 40% reduction in diagnostic costs is expected for specificity and per-patient test combinations ranging between 85%-$6 and 60%-$2. Triage tests with lower sensitivity could still be attractive in settings where the WTP threshold for health interventions is considerably lower than the WHO-CHOICE threshold of one GDP per capita [12], and to target populations with a lower TB prevalence for enhanced or active case finding.

To our knowledge no current TB tests have proven accuracy in a triaging algorithm and cost characteristics that fall within those ranges. A possible exception may be CXR in mass screening. The TB diagnostics development pipeline is filling up, and biomarker discovery projects are ongoing. Yet the ideal simple one-off highly sensitive and highly specific diagnostic test is not available. In the interim a test that could serve as a triage test may be a valuable spin-off.

While our examples are hypothetical, the modelling approach that we show could be useful when considering the feasibility of a potential triage test. High sensitivity is an important consideration. Triage tests with high sensitivity would have the advantage of a wide range of cost and specificity options that ensure the triage algorithm improves affordability, and may also avoid a costly tendency to add clinical diagnosis if the triage test result is negative. For the development of triage tests this implies that, if the technology and the costs allow, a cut-off value should be chosen that increases sensitivity at the expense of specificity, rather than choosing the optimal trade-off between sensitivity and specificity. Triage tests with lower sensitivity had a smaller affordability benefit over the Xpert-for-all algorithm, but very cheap tests may still be useful for resource limited settings where lower WTP thresholds apply in practice [12], and decision makers with very limited budgets have to choose between interventions with ICERS well below $100/DALY averted [27], [29]. In middle-income countries like South Africa Xpert is cost-effective for routine TB diagnosis [15]. In these settings triage tests would be useful as a screening tool for active case finding at lower TB prevalence while triage tests with lower sensitivity may not be attractive in passive case detection. Ultimately decision maker’s choices about affordability will be a trade-off between cost-effectiveness and other important decision-making factors that vary by country [12].

We showed that the specificity of a triage test is a trade-off with the per-patient test cost. In practice the cost to acquire and apply the device will vary by setting, by scale, and by technology depending on human resource, equipment and additional consumable requirements. For instance, the cost to apply a lateral flow device can be low in low-income countries. For rapid tests for detection of other infectious diseases, costs of $0.34 and $1.6 per patient tested in addition to the purchase of the test have been reported [26], [27]. For other technologies initial equipment cost may be considerable, so the per-patient test cost would largely depend on the expected number of patients that the equipment will be used for. An example of the latter is computer-aided reading of digital chest radiography (CAD) [30], an already available technology with very low running costs, of which the accuracy in a triage algorithm needs to be established [15].

In the future the cost for cartridge-based NAAT may reduce, or at least the cost for Xpert cartridges. Assuming the equipment and labour cost remain the same, even a cartridge cost of $3 would not achieve the same cost reduction as the triage algorithm in our primary analysis. The cost of cartridge-based real-time PCR technology is not likely to reduce to the levels of e.g. lateral flow assays and the current concessionary pricing for the Xpert cartridges (at $9.98 each [24]) is probably already close to the bottom price. Moreover, other elements contributing to the per-patient test cost (such as labor cost, cost for transporting, storing and discarding cartridges) are less variable. Therefore it is unlikely that per-patient costs for Xpert will come down to an extent that it much affects our conclusions.

Our analyses had several limitations. The model parameters were obtained from observations in health facilities and the analysis assumed that the triage and confirmatory test are performed at the same facility and same visit. The results are primarily applicable to improved case finding within health facilities (e.g. offering TB testing to all persons attending a health facility with any cough), or to congregational settings. We did in this analysis not consider operational aspects of test implementation. Additional diagnostic delays and drop-off between triage and confirmatory test were not included, and would lower the cost-effectiveness of a triage algorithm, similarly as would tests with lower sensitivity. When evaluating potential triage tests, those would have to be considered. Costs for outreach and transportation of samples or equipment were not considered, nor were possible effects on transmission; as far as effects on transmission can be expected our model is conservative. Screening and active case finding are increasingly encouraged as ways to improving stagnating trends in TB case detection [31]. A triage test would enhance the feasibility of screening, but the cost-effectiveness of TB screening and mass case finding would require further study and adjusted models.

For reasons of simplicity we assumed the sensitivity of the triage test to be relative to Xpert, thus reflecting a proportion of TB cases that can be detected by the confirmatory test. Assuming triage test sensitivity to be relative to culture and independent of Xpert, made the cost-effectiveness of triage algorithms look more favourable compared to Xpert-for-all. We assumed the accuracy to be independent of HIV status in the primary analysis and showed the direction of the effect if this were different. In practice the biological mechanism of a specific triage test will determine if and how the sensitivity of a triage tests interacts with Xpert and with HIV status. Overlap is expected when e.g. the same cases with a low bacillary concentration in sputum are missed by both tests. The results of these sensitivity analyses point out the importance of evaluating the accuracy of potential triage tests in combination with the presumed confirmatory tests, and in different sub-populations including HIV-positive and negative, early and advanced diseased. Assessing operational aspects of test implementation is an additional requirement before the cost-effectiveness of a specific triage test can be concluded. Our analysis aims to contribute to decisions about which tests or technologies may be worthwhile for further development and evaluation.

We did not include a base case of smear-microscopy followed by X-ray and clinical diagnosis if smears are negative [9], which in all three settings detected fewer TB cases than a triage algorithm based on a triage test with 70% relative sensitivity (data not shown). Lastly, our model does not consider the possibly higher MDR treatment cost as found in South Africa [28] nor the cost for HIV treatment [10] in the main analyses. Both would make algorithms with the highest case detection, i.e. Xpert-for-all less cost-effective, and thus make triage algorithms detecting fewer TB cases look more favourable.

## Conclusion

A triage test strategy to select persons for confirmatory testing with Xpert could substantially improve the affordability of Xpert for TB diagnosis. The affordability gain of a triage test with high sensitivity and modest specificity is particularly large with enhanced case finding. Further development of technologies that do not meet specification of a TB diagnostic POC, but may be suitable as a triage test, deserve encouragement.

## Supporting Information

Figure S1 **Cost-effectiveness plane comparing two diagnostic strategies: Xpert-for-all (Xpert) as the comparator and a triage algorithm (triage test, if positive followed by Xpert) as the base case.**
(TIF)

Figure S2 **Incremental Cost-Effectiveness Ratios (ICERs) of 'Xpert on all patients compared to triage algorithms for various sensitivity, specificity and cost combinations of a triage test in the India setting.**
(TIF)

Figure S3 **Incremental Cost-Effectiveness Ratios (ICERs) of 'Xpert on all patients compared to triage algorithms for various sensitivity, specificity and cost combinations of a triage test in the South Africa setting.**
(TIF)

Figure S4 **Acceptability cure comparing Xpert-for-all with a triage algorithm based on a triage test with 95% sensitivity, 75% specificity and per-patient test cost of $5, in the Uganda setting (GDP $487).**
(TIF)

Table S1 **Model inputs: cohort composition, diagnostic parameters and costs, by country setting.**
(DOCX)

Table S2 **Effect of adding clinical diagnosis in HIV-infected in the Uganda setting, on total cohort cost and costs per patient diagnosed by Xpert-for-all and Triage examples. For a cohort of 10 000 patients with presumed TB with 5% prevalence of smear-positive TB.**
(DOCX)

Methods S1 **METHODS expanded**
(DOCX)
