# Supplementary figures and images for: Optimal Triage Test Characteristics to Improve the Cost-Effectiveness of the Xpert MTB/RIF Assay for TB Diagnosis: A Decision Analysis
Source: PLoS One. 2013 Dec 18;8(12):e82786. doi: 10.1371/journal.pone.0082786 (PMC3867409; doi:10.1371/journal.pone.0082786)

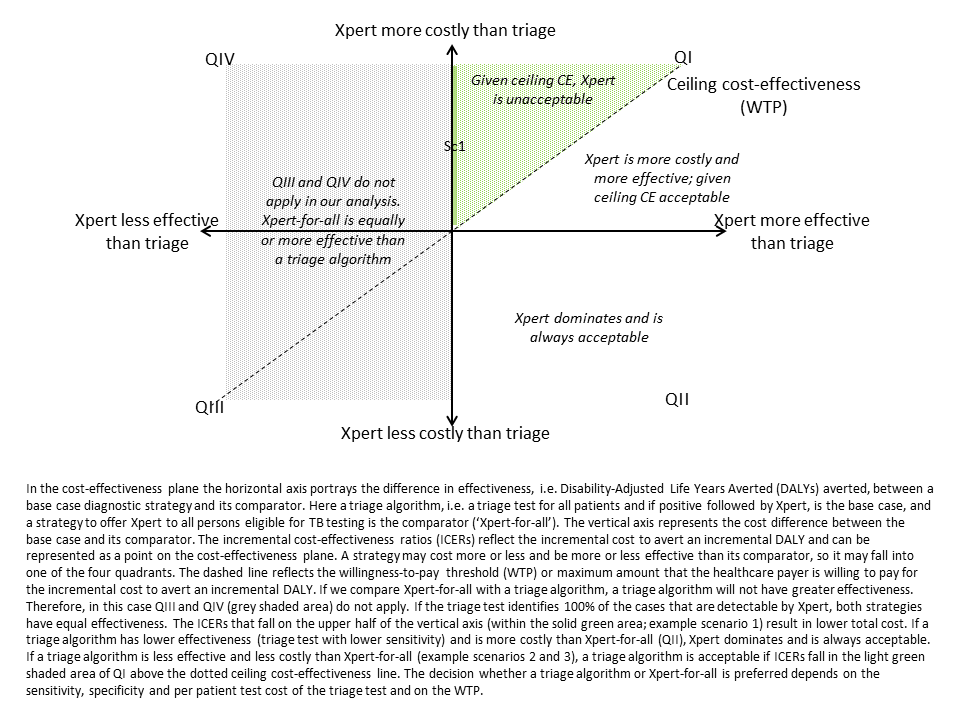

Supplement: Figure S1 — Cost-effectiveness plane comparing two diagnostic strategies: Xpert-for-all (Xpert) as the comparator and a triage algorithm (triage test, if positive followed by Xpert) as the base case. (TIF) [file pone.0082786.s001.tif]

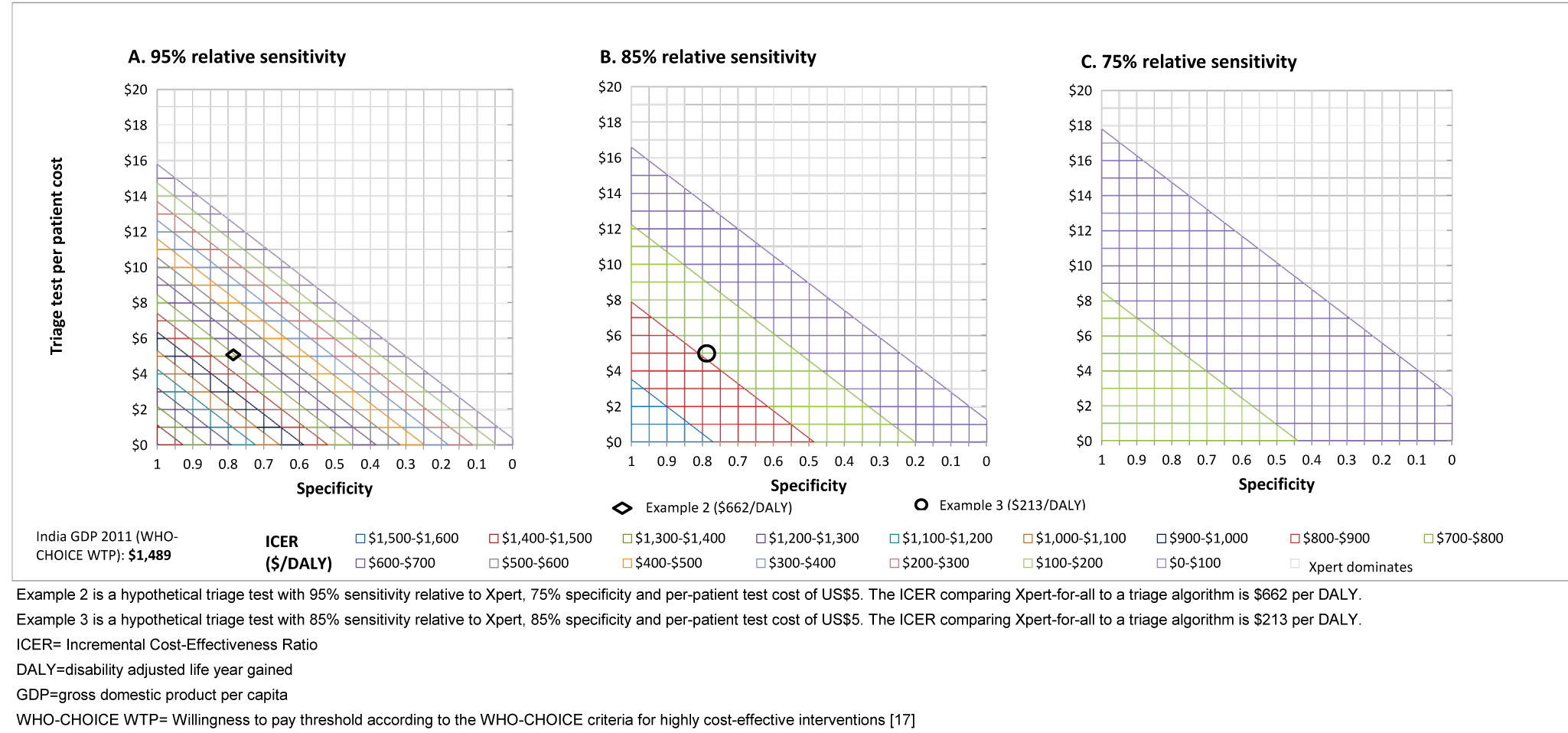

Supplement: Figure S2 — Incremental Cost-Effectiveness Ratios (ICERs) of 'Xpert on all patients compared to triage algorithms for various sensitivity, specificity and cost combinations of a triage test in the India setting. (TIF) [file pone.0082786.s002.tif]

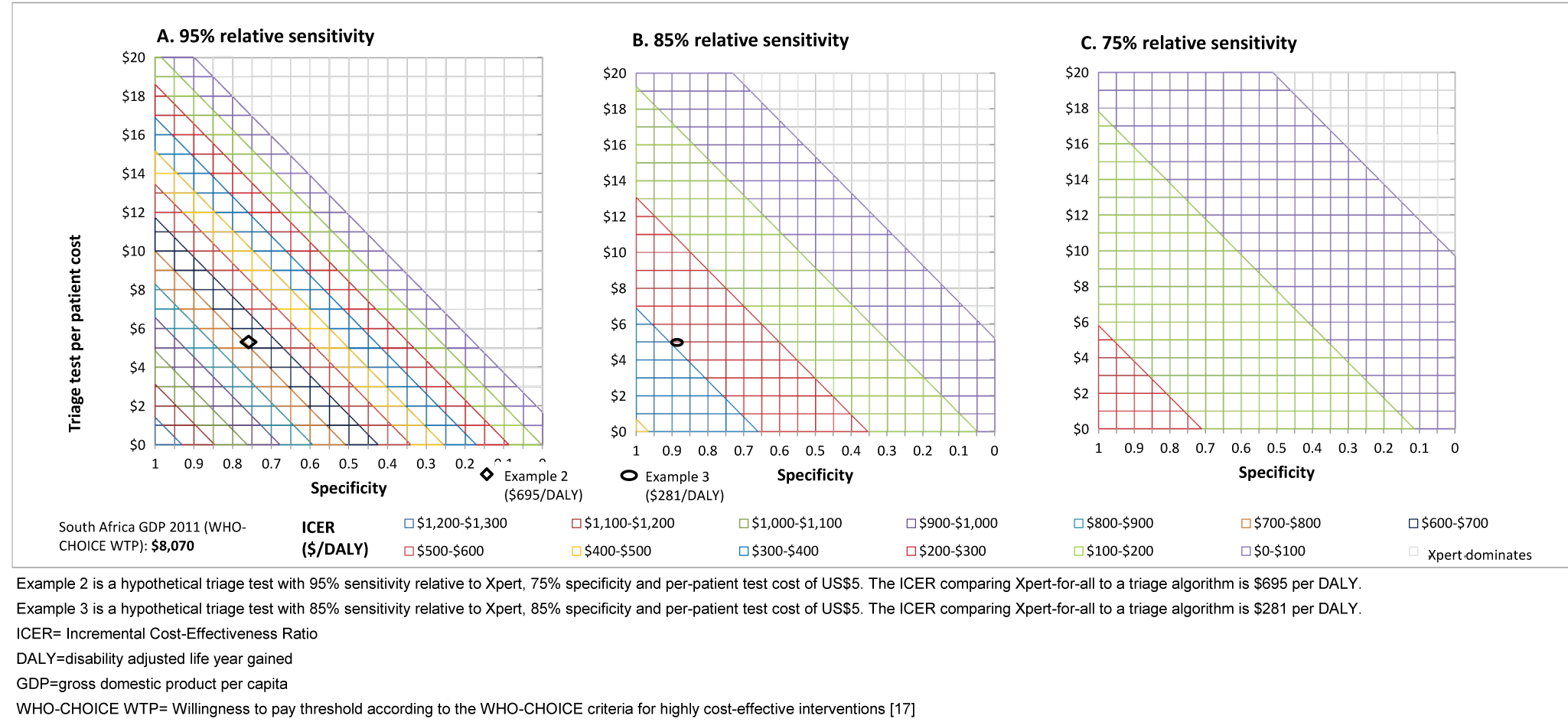

Supplement: Figure S3 — Incremental Cost-Effectiveness Ratios (ICERs) of 'Xpert on all patients compared to triage algorithms for various sensitivity, specificity and cost combinations of a triage test in the South Africa setting. (TIF) [file pone.0082786.s003.tif]

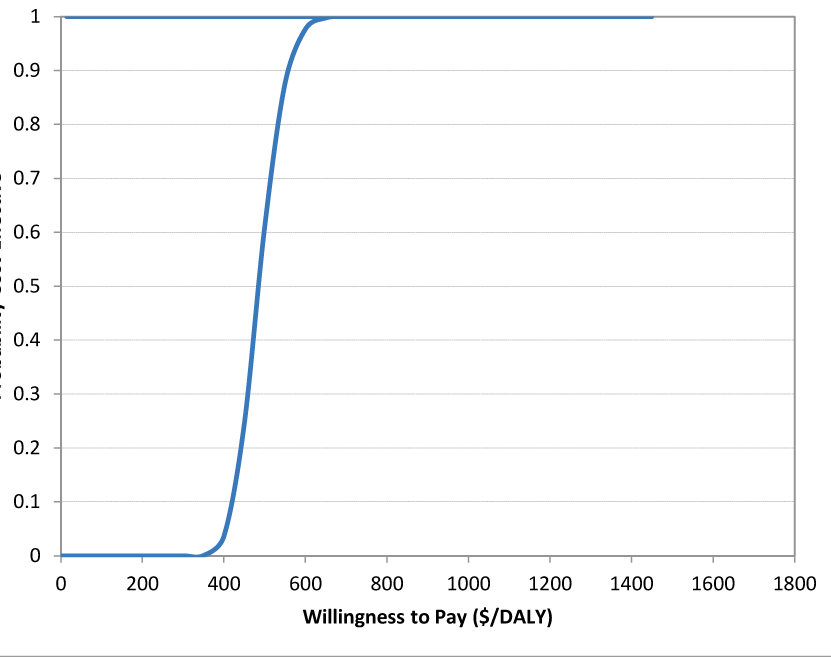

Supplement: Figure S4 — Acceptability cure comparing Xpert-for-all with a triage algorithm based on a triage test with 95% sensitivity, 75% specificity and per-patient test cost of $5, in the Uganda setting (GDP $487). (TIF) [file pone.0082786.s004.tif]
