# Supplementary material for: Optimal Triage Test Characteristics to Improve the Cost-Effectiveness of the Xpert MTB/RIF Assay for TB Diagnosis: A Decision Analysis
Source: PLoS One. 2013 Dec 18;8(12):e82786. doi: 10.1371/journal.pone.0082786 (PMC3867409; doi:10.1371/journal.pone.0082786)
Supplement: Table S1 — Model inputs: cohort composition, diagnostic parameters and costs, by country setting. (DOCX) [file pone.0082786.s005.docx]

Table S1. Model inputs: cohort composition, diagnostic parameters and costs, by country setting.

|  | **Uganda** | **India** | **South Africa** | **Distribution** | **Source** |
| --- | --- | --- | --- | --- | --- |
| **Cohort proportions** |  |  |  |  |  |
| Smear-positive TB | 0.05 | 0.05 | 0.05 | Beta | Model assumption |
| All TB (Smear-positive and Smear-negative)* | 0.091 | 0.069 | 0.091 | Beta | Demonstration study, all sites[2] |
| All TB among HIV-infected patients | 0.069 | 0.069 | 0.069 | Beta | Demonstration study, all sites[2] |
| All TB among non HIV-infected patients | 0.112 | 0.112 | 0.112 | Beta | Demonstration study, all sites[2] |
| Multidrug resistance among new TB cases | 0.011 | 0.023 | 0.066 | Beta | WHO[14] |
| Multidrug resistance among previously treated TB cases | 0.117 | 0.172 | 0.245 | Beta | WHO[14], survey[15] |
| Previous TB treatment among pulmonary TB cases | 0.073 | 0.192 | 0.168 | Beta | WHO[16] |
| HIV infection among presumptive and confirmed TB patients | 0.502 | 0.007 | 0.496 | Beta | WHO[16] |
| **Diagnostic parameters** |  |  |  |  |  |
| **Sensitivity for diagnosing PTB (SEM)** |  |  |  |  |  |
| Xpert MTB/RIF, smear-pos TB cases | 0.983 (0.005) | 0.983 (0.005) | 0.983 (0.005) | Beta | Demonstration study[2] |
| Xpert MTB/RIF, smear-neg TB cases HIV-neg | 0.793 (0.025) | 0.793 (0.025) | 0.793 (0.025) | Beta | Demonstration study[2] |
| Xpert MTB/RIF, smear-neg TB cases HIV-pos | 0.718 (0.040) | 0.718 (0.040) | 0.718 (0.040) | Beta | Demonstration study[2] |
| Mycobacterial culture* | 1 (--) | 1 (--) | 1 (--) | Beta | Model assumption |
| Clinical diagnosis† | 0.241 (0.032) | 0.241 (0.032) | 0.241 (0.032) | Normal | Demonstration study[2] |
| **Specificity for diagnosing PTB (SEM)** |  |  |  |  |  |
| Xpert MTB/RIF | 0.990 (0.002) | 0.990 (0.002) | 0.990 (0.002) | Beta | Demonstration study[2] |
| Mycobacterial culture** | 1 (--) | 1 (--) | 1 (--) | Uniform | Model assumption |
| Clinical diagnosis† | 0.942 (0.929, 0.952) | 0.942 (0.929, 0.952) | 0.942 (0.929, 0.952) | Triangular | Demonstration study[2] |
| **Sensitivity for detecting rifampicin-resistance (SEM)** | |  |  |  |  |
| Xpert MTB/RIF | 0.944 (0.015) | 0.944 (0.015) | 0.944 (0.015) | Beta | Demonstration study, all sites[2] |
| Conventional drug susceptibility testing | 1 (--) | -- | 1 (--) | -- | Model assumption |
| Line-probe assay | -- | 1 (--) | -- | -- | Model assumption |
| **Specificity for detecting rifampicin-resistance (SEM)** | |  |  |  |  |
| Xpert MTB/RIF | 0.983 (0.005) | 0.983 (0.005) | 0.983 (0.005) |  | Demonstration study, all sites[2] |
| Conventional drug susceptibility testing | 1 (--) | -- | 1 (--) | -- | Model assumption |
| Line-probe assay | -- | 1 (--) | -- | -- | Model assumption |
| **Diagnostic cost parameters** US$ 2011 (min, max) | |  |  |  |  |
| Xpert MTB/RIF§ | 19.23 (16.50, 21.97) | 14.01 (12.93, 15.10) | 17.00 (15.39, 18.61) | Triangular | Demonstration study[2]§ |
| Mycobacterial culture* | 16.10 (13.03, 19.16) | 14.07 (10.9, 17.28) | 16.95 (14.84, 19.05) | Triangular | Demonstration study[2] |
| Clinical diagnosis in TB positive patients | 2.02 (1.83, 2.21) | 5.35 (4.61, 6.08) | 9.29 (8.44, 10.14) | Triangular | Demonstration study[2] |
| Clinical diagnosis in TB negative patients | 2.05 (1.97, 2.12) | 4.01 (3.72, 4.30) | 8.69 (8.36, 9.03) | Triangular | Demonstration study[2] |
| **Treatment cost parameters** US$ 2011 (min, max) | |  |  |  |  |
| First-line category 1 treatment: total | 173 (114,232) | 236 (106,365) | 465 (306,624) | Triangular | WHO-CHOICE[17] literature review[18–22] |
| First-line category 2 treatment: total | 343 (226,461) | 365 (165,565) | 1091 (581,1601) | Triangular |  |
| Second-line treatment: total | 1804 (1294-2313) | 2338 (1516,3160) | 3618 (2143,5094) | Triangular |  |
| **DALY parameters:** DALYs averted (min, max) |  |  |  |  |  |
| HIV + SS- | 11.58  (10.63,12.90) | 9.38  (8.62, 10.39) | 10.71  (9.85,11.90) | Triangular | See Text Supplement |
| HIV - SS- | 18.65  (17.56, 19.61) | 13.18  (12.32, 13.96) | 13.83 (12.83,14.72) | Triangular | See Text Supplement |
| HIV + SS+ | 11.92  (10.63, 12.90) | 9.67  (8.62, 10.39) | 11.03 (9.85,11.90) | Triangular | See Text Supplement |
| HIV - SS+ | 22.63  (22.13, 23.07) | 16.43  (16.02, 16.79) | 17.52  (17.05, 17.93) | Triangular | See Text Supplement |

SEM=standard error of the mean

neg=negative pos=positive

**The prevalence of pulmonary TB in each epidemiological setting was derived from (i) the prevalence of smear-positive TB among the patients, and from (ii) the prevalence of HIV among TB cases and the relationship between the number of smear-positive and smear-negative cases among the patient cohort, taken as a fixed ratio depending on the patient’s HIV infection status (0.72 in HIV-positive, 0.45 in HIV-negative patients), as observed in the demonstration study [2].

*LJ culture in Uganda and India, Mycobacterium Growth Inhibitor Tube (MGIT) in South Africa

†Average of observations in the three country settings

§Includes US$ 9.98 per cartridge cost (subsidized pricing[23]), and 2.4% repeats for indeterminate results as observed in the demonstration study.
