# Supplementary material for: Optimal Triage Test Characteristics to Improve the Cost-Effectiveness of the Xpert MTB/RIF Assay for TB Diagnosis: A Decision Analysis
Source: PLoS One. 2013 Dec 18;8(12):e82786. doi: 10.1371/journal.pone.0082786 (PMC3867409; doi:10.1371/journal.pone.0082786)
Supplement: Table S2 — Effect of adding clinical diagnosis in HIV-infected in the Uganda setting, on total cohort cost and costs per patient diagnosed by Xpert-for-all and Triage examples. For a cohort of 10 000 patients with presumed TB with 5% prevalence of smear-positive TB. (DOCX) [file pone.0082786.s006.docx]

Table S2 - Effect of adding clinical diagnosis in HIV-infected in the Uganda setting, on total cohort cost and costs per patient diagnosed by Xpert-for-all and Triage examples. For a cohort of 10 000 patients with presumed TB with 5% prevalence of smear-positive TB.

| Scenario | Clinical diagnosis in HIV+ | Number of suspects in cohort with TB | Total TB case detected | % of TB cases detected | Cohort Diagnostic Costs (US$2011) | Increase in diagnostic cost | Diagnostic cost per TB case detected (US$ 2011) | Treatment Costs (US$2011) | Total Costs ($2011) | Increase in total cost | Total cost per TB case detected (US$ 2011) | Increase in cost per TB case detected |
| --- | --- | --- | --- | --- | --- | --- | --- | --- | --- | --- | --- | --- |
| Xpert for all patients | None | 959 | 912 | 95% | 195294 | Baseline | 214 | 213602 | 408895 | Baseline | 448 | Baseline |
|  | after neg Xpert | 959 | 927 | 97% | 206278 | 6% | 223 | 274712 | 480990 | 18% | 519 | 16% |
| Triage example 2. 95% sensitivity, 75% specificity, cost $5 | None | 959 | 895 | 93% | 112204 | Baseline | 125 | 197046 | 309250 | Baseline | 345 | Baseline |
|  | after neg Xpert | 959 | 895 | 93% | 114996 | 2% | 128 | 211779 | 326776 | 6% | 365 | 6% |
|  | after neg Xpert and after neg triage test | 959 | 915 | 95% | 123239 | 10% | 135 | 259387 | 382626 | 24% | 418 | 21% |
| Triage example 3. 85% sensitivity, 85% specificity, cost $5 | None | 959 | 853 | 89% | 94299 | Baseline | 111 | 186160 | 280460 | Baseline | 329 | Baseline |
|  | after neg Xpert | 959 | 853 | 89% | 95975 | 2% | 113 | 195000 | 290975 | 4% | 341 | 4% |
|  | after neg Xpert and after neg triage test | 959 | 884 | 92% | 105322 | 12% | 119 | 250605 | 355928 | 27% | 403 | 22% |
