# Supplementary material for: Optimal Triage Test Characteristics to Improve the Cost-Effectiveness of the Xpert MTB/RIF Assay for TB Diagnosis: A Decision Analysis
Source: PLoS One. 2013 Dec 18;8(12):e82786. doi: 10.1371/journal.pone.0082786 (PMC3867409; doi:10.1371/journal.pone.0082786)
Supplement: Methods S1 — METHODS expanded (DOCX) [file pone.0082786.s007.docx]

**SUPPORTING INFORMATION**

**METHODS expanded**

We modified the decision analytical model used for an economic evaluation of Xpert, which has been described in detail elsewhere [1]. The model was parameterized (Table S1) with data from a multi-country demonstration study of this diagnostic in three epidemiological and economic settings: India (low HIV prevalence, low MDR prevalence), Uganda (high HIV prevalence, low MDR prevalence), and South Africa (high HIV prevalence, moderately high MDR prevalence) [2]. The model follows a cohort of 10 000 individuals presumptive TB and requiring diagnostic testing through the diagnostic and treatment pathway, estimating costs and health gains. The outcomes were total costs, divided into treatment costs and test costs, DALYs averted, TB cases detected and MDR-TB cases detected. Only health care-related costs are taken into account.

For both algorithms TB cases were characterized in the model as: (i) new or previously treated, (ii) HIV-negative or HIV-positive, (iii) multi-drug resistant (MDR) or drug susceptible, and (iv) as smear-positive or smear-negative, to accommodate differences in sensitivity of Xpert by smear status [2]. We assumed that the presence of rifampicin resistance conferring mutations implied the need for 2^nd^ line treatment [3]. The sensitivity of the tests in the algorithms determined the probability that TB cases among the cohort were diagnosed as having TB. The specificity determined the probability that those without TB were diagnosed as having TB. In both algorithms TB cases who remained undiagnosed due to negative results of Xpert or the triage test, respectively, return for re-testing after three months unless they die or self-cure within that time. Of the smear-negative TB cases that remained undiagnosed and returned to the clinic after 3 months 10% were assumed to have become smear-positive within that time. True negative patients (i.e. who have no TB and were not diagnosed with TB) do not return for diagnosis. Model assumptions with regard to HIV and to diagnosis and treatment of drug-resistant TB were maintained [1].

The model input parameters (Table S1) were maintained from the published model[1] with respect to the prevalence in the cohort of prior TB treatment, HIV, multi-drug resistance (MDR), and smear-positivity to accommodate differences in sensitivity of Xpert by smear status [2], and model assumptions with regard to HIV and diagnosis and treatment of drug-resistant TB. Treatment outcome probabilities and disability adjusted life years (DALYs) averted for patients being cured were also maintained.[1] They originated from published meta-analyses of clinical trials, cohort studies, and systematic reviews [4–12], and DALYs were estimated using the standard formula [13].

The prevalence of pulmonary TB in each epidemiological setting (Table S1) was derived from the prevalence of smear-positive TB among the patients with presumed TB, taken as a fixed ratio depending on the patients’ HIV infection status (0.72 in HIV-positive, 0.45 in HIV-negative patients), as observed in the demonstration study [2]. Diagnostic costs were collected at the demonstration sites using the ingredient costing approach [1]. All local costs were reported in 2011 US$ and converted using the average exchange rate for 2011 (imfstatext.imf.org). Where relevant, costs were annualized using a standard discount rate of 3% [13].

**Supplement** Reference List

1. Vassall A, van Kampen S., Sohn H, Michael JS, John K, et al. (2011) Rapid diagnosis of tuberculosis with the Xpert MTB/RIF assay in high burden countries: a cost-effectiveness analysis. PLoS Med 8: e1001120. 10.1371/journal.pmed.1001120 [doi];PMEDICINE-D-11-00805 [pii].

2. Boehme CC, Nicol MP, Nabeta P, Michael JS, Gotuzzo E, et al. (2011) Feasibility, diagnostic accuracy, and effectiveness of decentralised use of the Xpert MTB/RIF test for diagnosis of tuberculosis and multidrug resistance: a multicentre implementation study. 377: 1495-1505.

3. World Health Organization (2011) Guidelines for the programmatic management of drug-resistant tuberculosis. http://whqlibdoc/ who int/publications/2011/9789241501583_eng pdf .

4. Abdool Karim SS, Naidoo K, Grobler A, Padayatchi N, Baxter C, et al. (2010) Timing of initiation of antiretroviral drugs during tuberculosis therapy. N Engl J Med 362: 697-706. 362/8/697 [pii];10.1056/NEJMoa0905848 [doi].

5. Akksilp S, Karnkawinpong O, Wattanaamornkiat W, Viriyakitja D, Monkongdee P, et al. (2007) Antiretroviral therapy during tuberculosis treatment and marked reduction in death rate of HIV-infected patients, Thailand. Emerg Infect Dis 13: 1001-1007. 10.3201/eid1307.061506 [doi].

6. Espinal MA, Kim SJ, Suarez PG, Kam KM, Khomenko AG, et al. (2000) Standard short-course chemotherapy for drug-resistant tuberculosis: treatment outcomes in 6 countries. JAMA 283: 2537-2545. joc92107 [pii].

7. Lew W, Pai M, Oxlade O, Martin D, Menzies D (2008) Initial drug resistance and tuberculosis treatment outcomes: systematic review and meta-analysis. Ann Intern Med 149: 123-134. 149/2/123 [pii].

8. Menzies D, Benedetti A, Paydar A, Royce S, Madhukar P, et al. (2009) Standardized treatment of active tuberculosis in patients with previous treatment and/or with mono-resistance to isoniazid: a systematic review and meta-analysis. PLoS Med 6: e1000150.

9. Nathanson E, Lambregts-van WC, Rich ML, Gupta R, Bayona J, et al. (2006) Multidrug-resistant tuberculosis management in resource-limited settings. Emerg Infect Dis 12: 1389-1397. 10.3201/eid1209.051618 [doi].

10. Seung KJ, Omatayo DB, Keshavjee S, Furin JJ, Farmer PE, et al. (2009) Early outcomes of MDR-TB treatment in a high HIV-prevalence setting in Southern Africa. PLoS One 4: e7186. 10.1371/journal.pone.0007186 [doi].

11. Varma JK, Nateniyom S, Akksilp S, Mankatittham W, Sirinak C, et al. (2009) HIV care and treatment factors associated with improved survival during TB treatment in Thailand: an observational study. BMC Infect Dis 9: 42. 1471-2334-9-42 [pii];10.1186/1471-2334-9-42 [doi].

12. Wells CD, Cegielski JP, Nelson LJ, Laserson KF, Holtz TH, et al. (2007) HIV infection and multidrug-resistant tuberculosis: the perfect storm. J Infect Dis 196 Suppl 1: S86-107. JID38320 [pii];10.1086/518665 [doi].

13. Tan-Torres Edejer, T., Baltussen, R., Ad, , T, Hutubes, , R., Achar, , A., et al. (2003) Making choices in health: WHO guide to cost-effectiveness analysis. World Health Organization, 20 Avenue Appia, 1211 Geneva 27, Switzerland.

14. World Health Organization (2013) Surveillance of drug resistance in tuberculosis. http://who.int/tb/publications/mdr_surveillance/en/index.html.

15. Lukoye D, Cobelens FG, Ezati N, Kirimunda S, Adatu FE, et al. (2011) Rates of anti-tuberculosis drug resistance in Kampala-Uganda are low and not associated with HIV infection. PLoS One 6: e16130. 10.1371/journal.pone.0016130 [doi].

16. World Health Organization (2010) global tuberculosis control: who report 2010.

17. World Health Organization (2012) CHOosing Interventions that are Cost Effective (WHO-CHOICE). http://www.who.int/choice/en/.

18. Floyd K, Arora VK, Murthy KJ, Lonnroth K, Singla N, et al. (2006) Cost and cost-effectiveness of PPM-DOTS for tuberculosis control: evidence from India. Bull World Health Organ 84: 437-445. S0042-96862006000600012 [pii];/S0042-96862006000600012 [doi].

19. Okello D, Floyd K, Adatu F, Odeke R, Gargioni G (2003) Cost and cost-effectiveness of community-based care for tuberculosis patients in rural Uganda. Int J Tuberc Lung Dis 7: S72-S79.

20. Pantoja A, Lonnroth K, Lal SS, Chauhan LS, Uplekar M, et al. (2009) Economic evaluation of public-private mix for tuberculosis care and control, India. Part II. Cost and cost-effectiveness. Int J Tuberc Lung Dis 13: 705-712.

21. Sinanovic E, Floyd K, Dudley L, Azevedo V, Grant R, et al. (2003) Cost and cost-effectiveness of community-based care for tuberculosis in Cape Town, South Africa. Int J Tuberc Lung Dis 7: S56-S62.

22. Sinanovic E, Kumaranayake L (2006) Financing and cost-effectiveness analysis of public-private partnerships: provision of tuberculosis treatment in South Africa. Cost Eff Resour Alloc 4: 11. 1478-7547-4-11 [pii];10.1186/1478-7547-4-11 [doi].

23. Stop TB Department (2012) Buy-down agreement to reduce cost of Xpert MTB/RIF cartridges by 40% for high-burden countries. http://www stoptb org/wg/new_diagnostics/assets/documents/News_XpertPrice_21Aug12 pdf .
